# Towards Remote Lightning Manipulation by Meters-long Plasma Channels Generated by Ultra-Short-Pulse High-Intensity Lasers

**DOI:** 10.1038/s41598-018-36643-2

**Published:** 2019-01-23

**Authors:** Jenya Papeer, Indranuj Dey, Moti Botton, Zohar Henis, Amit D. Lad, Moniruzzaman Shaikh, Deep Sarkar, Kamalesh Jana, Sheroy Tata, Sudipta Lodh Roy, Yash M. Ved, G. Ravindra Kumar, Arie Zigler

**Affiliations:** 1 Racah Institute of Physics, The Hebrew University of Jerusalem, Jerusalem, 91904 Israel; 2 Tata Institute of Fundamental Research, 1 Homi Bhabha Road, Colaba, Mumbai, 400005 India

## Abstract

Remote manipulation (triggering and guiding) of lightning in atmospheric conditions of thunderstorms has been the subject of intense scientific research for decades. High power, ultrashort-pulse lasers are considered attractive in generating plasma channels in air that could serve as conductors/diverters for lightning. However, two fundamental obstacles, namely the limited length and lifetime of such plasma channels prevented their realization to this date. In this paper, we report decisive experimental results of our multi-element broken wire concept that extends the generated plasma channels to the required tens of meters range. We obtain 13-meter-long plasma wire, limited only by our current experimental setup, with plasma conditions that could be sufficient for the leader initiation. This advance, coupled with our demonstrated method of laser heating for long time sustenance of the plasma channel, is a major, significant step towards controlling lightning.

## Introduction

Remote manipulation - triggering and guiding of lightning in atmospheric conditions of thunderstorms has been the objective of intense scientific research for the last four decades due to its huge importance for human protection, averting damages to vital installations and safeguarding the economy. Earliest optically based schemes utilized high energy CO_2_ lasers since they were powerful enough to ionize air and provide a preferential path for lightning^1^. This concept was later abandoned due to difficulties in focusing the laser at distances of several hundreds of meters and the tendency of the laser to produce discrete, localized ionization points (ionized dots) rather than long and continuous plasma channels. The advent of ultrashort (sub-ps) pulsed high-intensity (above 1TW) lasers (employing Chirped Pulse Amplification - CPA^2^) and their ability to produce meter-long, short lifetime channels in air has provided an impetus for reconsideration of an all-optical approach of achieving that goal^3,4^. A femtosecond high-intensity laser pulse undergoing filamentation^5,6^ can create a smooth plasma channel at its wake. This plasma channel in principle can be considered as an artificially created conducting zone that can replace the metallic wire used in some non-optical schemes of lightning manipulation.

As noted by Bazelyann and Raizer^7^, the formation of twin leaders - often referred to as bi-directional leaders - is a key element of artificial lightning discharges. Remote artificial triggering of lightning by a laser, therefore, requires the formation of one of the twin leaders by a laser-induced plasma channel that plays the role of the conductor. This requires plasma with high enough conductivity (electron density above 10^15^ cm^−3^), slow recombination time to provide enough time for the lightning to discharge, and sufficient length (exceeding 20 m) (see an estimate in methods). Plasma channels in air are characterized by a far lower initial conductivity and shorter lifetime than the required parameters. Moreover, Eisenmann *et al*.^8^ showed that the length of a plasma filament generated by ultrashort pulsed laser is limited to a meter or so by the amount of energy confined in the filament and the capability to replenish it from the surrounding laser beam energy^9^. Finally, when the energy contained in the laser pulse exceeds, by many times, the critical power required for self-focusing, the laser beam breaks into several randomly generated filaments, where the number and position vary from shot to shot (for example, see refs^10,11^). Consequently, these properties of the straight-forward generated plasma filaments have been the major impediments to previous efforts to trigger lightning in the atmosphere^12,13^, and thus, despite considerable efforts^14–17^ a direct implementation of this concept is yet to demonstrate electrical guiding over significant distances.

Over the years research has been carried out in order to overcome these major obstacles^18–20^. Addressing the short lifetime of the plasma channel, a dual laser system that combines an Ultra-Short Laser Pulse to initiate air breakdown and create plasma channels followed by a high energy Long Pulse Laser (ns time duration) to maintain the required high conductivity plasma was proposed^21^. A proof of principles of that approach was indeed recently reported^22–24^. A scheme has been developed^25^ to control the number of filaments and filamentation position using a double-lens setup with a defocusing lens followed by a focusing lens. By varying the distance between the two lenses a continuous control of the position of the filamentation collapse between 12 m to 400 m was demonstrated. Although these two innovations addressed major difficulties in the realization of the laser-based plasma channel in lightning manipulations, there remained one major obstacle, specifically, the length of the produced channel. Gaining control over the position of filamentation, as well as removing the erratic breakup of the filament, paved the way to another innovative approach, namely, concatenating several filaments in order to form a broken-wire which produces a longer effective wire. A proof of principle of the broken-wire approach was recently demonstrated^26^.

The main objective of this paper is to provide and experimentally verify the missing fundamental step required for remote triggering and guiding of lightning discharges by using laser-produced plasma channels, specifically, generation of extended-length plasma channel. We demonstrate generation of a 13-meter long filament (limited only by our current experimental set up), which is an order of magnitude increase over than those achieved previously. This multi-broken-wire filament is obtained using a single ultra-short laser pulse. We demonstrate an accurate control over the initial position of each element of the multi-broken-wire channel. Moreover, since lateral position of each filament is slightly shifted across the laser beam cross-section and exposed to a different part of the laser beam it can be maintained by the laser energy intercepting that particular area. The distance between consecutive plasma fragments is a few millimeters and can easily be bridged by the propagating electrical discharge^20^. As there is no fundamental limit on the multi-broken-wire scheme, this demonstrated 13 m long plasma channel can be extended to a much longer wire given an improved laser and optical system. We present calibrated^22–24^ numerical simulations for the heating of the “multi-broken wire” plasma channel resorting to a Nd:YAG (*λ* = 1.064 µm) and a CO_2_ (*λ* = 9–12 µm) lasers and evaluate the feasibility of remote heating and maintaining the generated multi-broken-wire channel for a longer time. Our study explores the physics that will make remote manipulation of lightings feasible in the near future.

## Results

### Broken-wire generation

The experiment was performed with the 100 TW laser facility at the Ultrashort Pulse High Intensity Laser Laboratory (UPHILL) in the Tata Institute of Fundamental Research (TIFR), Mumbai. The laser has a central wavelength of 800 nm, with a pulse width of ~50 fs (10 Hz repetition rate) in air and a beam diameter of ~80 mm. The output energy from the laser (after compression) was limited to ≤100 mJ to prevent filamentation in windows and lenses employed in the experiment. We note that the laser power actually used during the experiment was 2–3TW.

**Figure 1:**
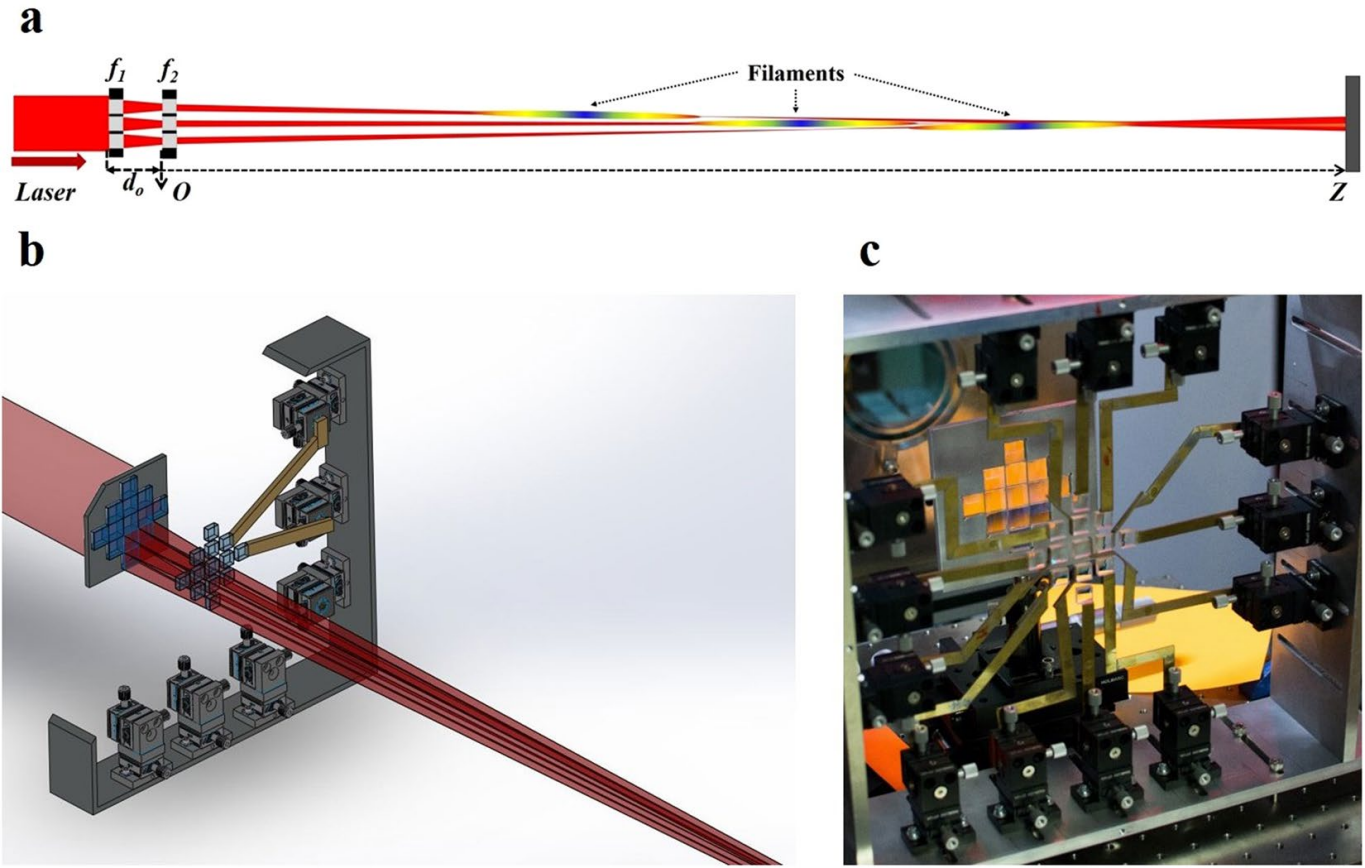
Optical assembly for the generation of Broken Wire. (**a**) Planar schematic of the setup showing 3 lenses and the concept of broken wire filaments. (**b**) 3D schematic of the lens assembly. The XYZ actuators (part number DT12XYZ) drawing is from Thorlabs Inc. The schematic is simplified by partial representation of the assembly and the optical rays. (**c**) A photograph of the experimental setup during the experiment.

The experimental setup schematic for the controlled generation of the broken wire plasma channel is shown in Fig. 1(a). It consists of a tightly packed array of double-lens telescopic systems. Each telescope is designed to separately focus a different region of the laser beam in order to generate a stable plasma channel (fragment of the “broken wire”) with high accuracy in its position and shot to shot repeatability. Every telescope consists of converging and diverging lens separated by a distance *d*_0_. The converging lenses are assembled in a tightly packed, static array while the diverging lenses are slightly separated and individually mounted on XYZ actuators. Figure 1(b) shows a photograph of the converging lens array (L_AC_) and diverging lens array (L_AD_) consisting of 13 lenses each, in the assembled condition during the experiment. The lenses are arranged in a cross pattern, covering ~80 mm along the horizontal and vertical axis [see Fig. 1]. The focal length of the converging lenses is ${f}_{1}=\,20\,{cm}$, while that of the diverging lenses is ${f}_{2}=-\,10\,{cm}$, and the distance between them is ${d}_{0}\approx 10\,{cm}$. The lenses have a rectangular shape, to enable tight packing of the first lens array and avoid losing any laser radiation to the slits between lenses. The total power transmission of the optical system was above 90%. Each diverging lens in the array is individually controllable in the three Cartesian axes (Z-axis is aligned with the laser propagation direction), giving control over the spatial position of each lens [see Fig. 1(a)] both in the X-Y plane and the Z-direction. The use of converging, followed by a diverging lens is crucial for allowing the mechanical assembly of the controlled (second) array. The dimensions of the lenses are 1.6 × 1.6 cm for the first lens and 1.1 × 1.1 cm for the second lens, allowing a gap of 0.5 cm between the lenses in the second array. This gap enables to connect each lens to an actuator using a thin copper strip and keeps enough space for efficient movement of each lens in the X-Y plane.

**Figure 2:**
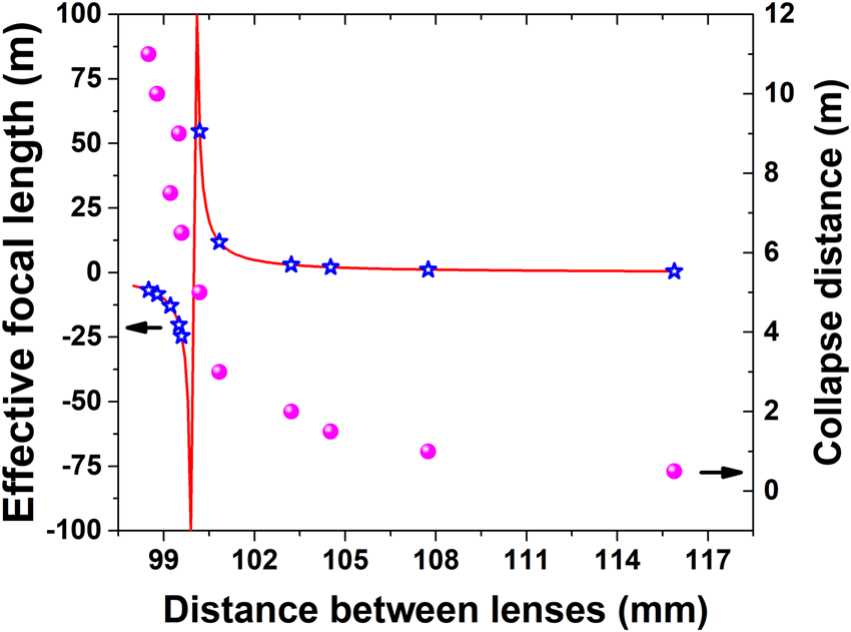
Effective focal length estimation. Calculation of the focal/collapse distance of a telescope as a function of *d*_0_ (the distance between the lenses). The selected values of *d*_0_ correspond to the actual distances between the converging and the diverging lenses used in the experiment. Right axis (circles) using the BFL and Marburger’s equation and the method described in Eisenmann *et al*.^25^ Left axis (solid line and stars) using only the BFL equation.

The effective focal length of a telescopic lens system is given by ${f}_{eff}={f}_{2}({d}_{0}-{f}_{1})/\{{d}_{0}-({f}_{1}+{f}_{2})\}$ (this equation is known as the Back Focal Length (BFL) equation describing the distance of the focal point from the second (diverging) lens). Therefore, the focal length for each pair of lenses in the array can be varied over a distance of 15 m, by changing *d*_0_ by ~5 mm, as shown in Fig. 2 (left axis). By appropriately adjusting *d*_0_, individual filaments can be created at different distances along the laser propagation axis. The initial evaluation of the required distances between the lenses (*d*_0_) was done by calculating the collapse distance of a beam propagating through an optical setup using the Marburger’s equation^27^ and a method described in ref ^25^. The outcome of that calculation is presented in Fig. 2 (right axis). This calculated collapse distance depends on the power of the corresponding laser beam and the focal length of the corresponding optical system (the BFL). The calculation was done by substituting the actual values of *d*_0_ (and the corresponding BFL calculation) and the optical power used in the experiment. The left axis on Fig. 2 presents the calculated BFL (solid line) while the stars note the substitution of the actual distance between the two lenses (i.e. *d*_0_) used in the experiment. The first datapoint (smallest *d*_0_) corresponds to the furthest filament and so on. It demonstrates that the optical configuration used for the last 5 filaments was that of a diverging telescope which delays the natural collapse distance of the beam. On the other hand, the first 6 filaments were focused by a converging telescope configuration. Figure 2 demonstrates the required sensitivity of *d*_0_ alignment for controlling the position of each filament. The position of the focus (and therefore the filament) of each pair of lenses in the plane perpendicular to the propagation direction can be controlled by manipulating the diverging (i.e. second) lens position in the X-Y plane.

**Figure 3:**
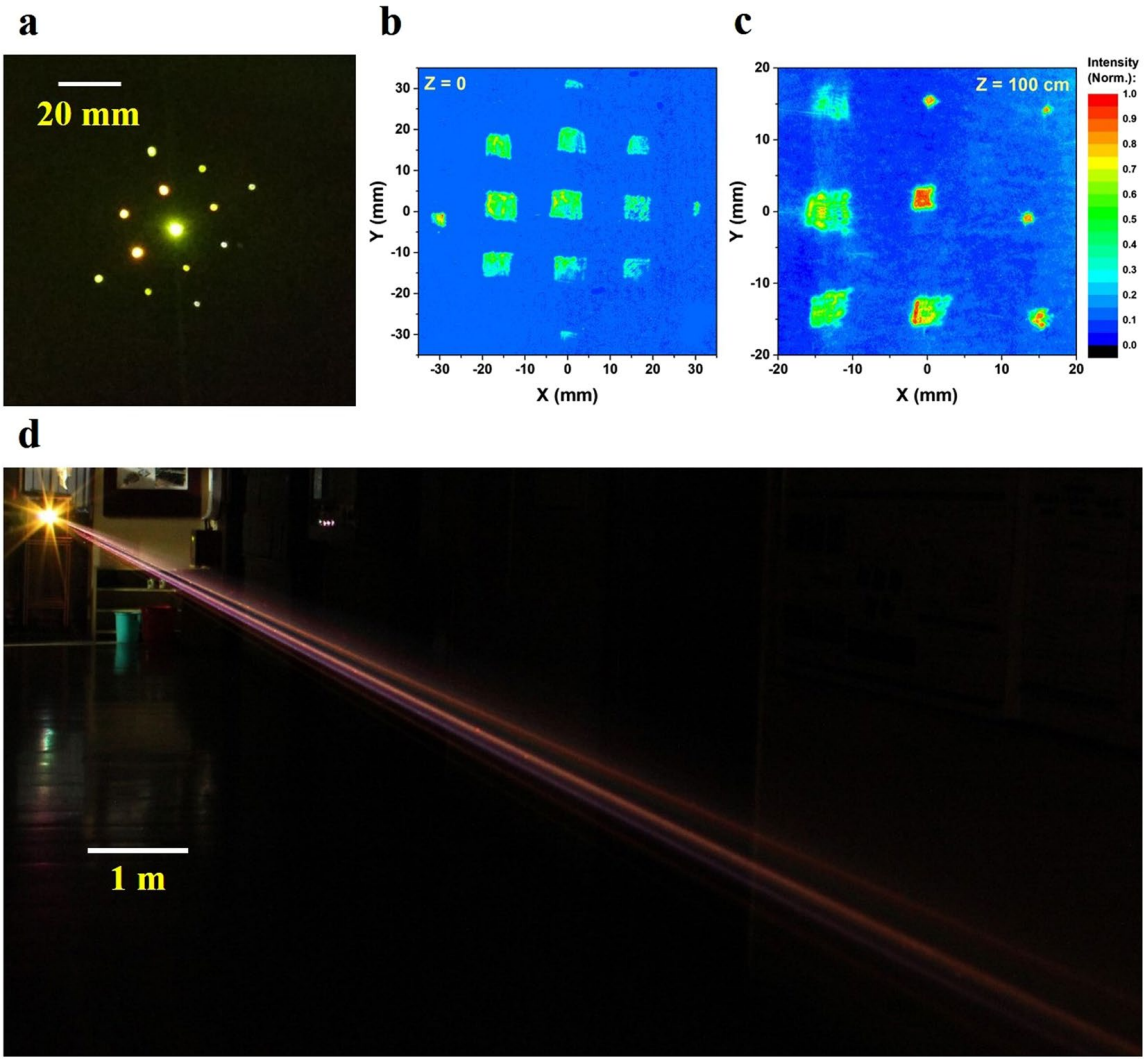
Photographs of the broken wire filaments. (**a**) A picture of the focal plane of the 13 beams, aligned to a distance of 2 m from the optical setup. An image of the beam profile during the experiment, (**b**) after the optical setup, and (**c**) at a distance of 1 m from it. (**d**) A picture of the Broken Wire, taken ~12 meters from the optical setup during the experiment.

The alignment of the optical system during the experiment was carried out in three steps. In the first step, all the lenses in the diverging lens array were positioned at same *d*_0_ (~11 cm), so that the collapse distance was ~2 m from the diverging array. In the next step, the X-Y position of each diverging lens was adjusted so that the beamlets incident on the array were centered on each lens, and the outcoming beams would be parallel to each other, this was assured by observing the focused beam profile close to the collapse distance, as demonstrated in Fig. 3(a). In the final step, the distance *d*_0_ was fine-tuned between each pair of converging-diverging lenses to move the beam collapse distances along the Z axis. Using this procedure allowed achieving the desired arrangement of the broken wire fragments. Each consecutive plasma filament was aligned to initiate immediately after the plasma density of the previous channel dropped below 10^15^ cm^−3^.

Figure 3(a) shows a photograph of the light emitted from filament array formed by the telescopic system on a fluorescent paper near the optical setup. Figure 3(b,c) demonstrates the optical profile of the beam immediately after the optical setup and at a distance of 1 meter from it, respectively. It is clearly seen that each beam is being focused differently. The beam in the upper-right corner [Fig. 3(c)], for example, is being almost focused and is on the onset of collapsing into a plasma filament while the beam in the upper-left corner is being weakly diverged, to collapse at a longer distance. This optical assembly allows an individual and accurate control over 13 fragments of the beam. By mounting the diverging lenses on XYZ stages with micrometric actuators we were able to control the position of the focal spot of each fragment of the beam with an accuracy of ~1 mm both in the XY plane and the propagation direction. Though the individual beamlets were positioned in an array to demonstrate the differences in collapse distance, they can be brought close to each other using the XY control on the diverging lenses to lie almost collinear with respect to each other, giving rise to a quasi-continuous broken wire channel. Figure 3(d) shows the filament array in a corridor adjacent to the laser facility in TIFR. The photograph was obtained with a long exposure (~30 s) at 1600 ISO using a Canon 1300D camera. The visible streaks are due to the scattering of the supercontinuum originated from the laser pulse undergoing filamentation by dust particles.

### Plasma density measurements

**Figure 4:**
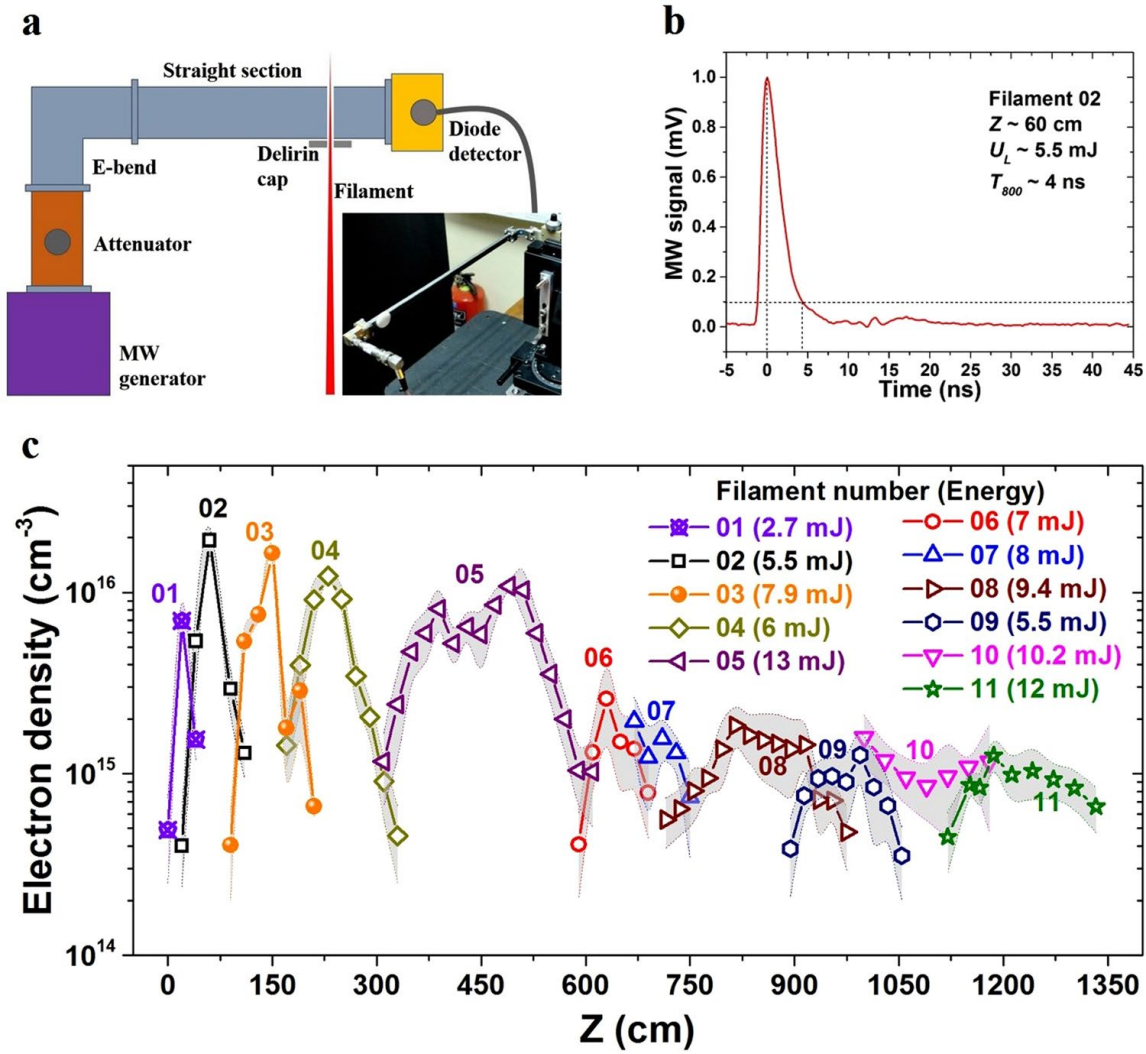
Measurement of plasma density along the broken wire channel. (**a**) A sketch and an image of the microwave diagnostics setup. (**b**) A typical measurement of plasma evolution obtained by the microwave setup, (**c**) Plasma density along the broken wire channel. Each curve represents a different fragment of the beam, the energy of laser radiation in each fragment is indicated in the legend of the figure. The shade over the data points represents the error of the measurement.

The temporal evolution of the filament plasma electron density (and hence the filament lifetime) was recorded by capturing the attenuation in a K_a_-band microwave (MW) transmission waveguide (26.5–40 GHz) caused by the filament traversing the waveguide perpendicular to its microwave propagation axis (described in detail in earlier works)^28,29^. In the current work, a new compact MW setup has been used with several advantages over the earlier microwave set up. This includes a Gunn-diode with higher frequency (37 GHz) output, which gives better space and time resolution. The output microwave power is higher (~200 mW) for better signal to noise ratio. A high-quality isolator is incorporated to minimize successive reflection of MW signals in the waveguide. The schematic of the microwave diagnostic setup is shown in Fig. 4(a) along with a picture of the assembly in the inset. Figure 4(b) shows a typical signal from the MW setup for a filament generated by a 5.5 mJ beam, demonstrating a lifetime of ~3 ns, consistent with results from our previous works^22,23,29^. The maximum plasma density of the filament was found to be ~10^16^ cm^−3^, which was obtained by comparing the e-folding time/decay time of the experimental signal with the simplified relaxation rate equations presented below^29^

1 $$\frac{d{n}_{e}}{dt}=-\,\beta {n}_{e}{n}_{{O}_{2}^{+}}-\eta {n}_{e}-\gamma {n}_{{O}_{4}^{+}}{n}_{e}$$

2 $$\frac{d{n}_{{O}_{2}^{+}}}{dt}=-\,\beta {n}_{e}{n}_{{O}_{2}^{+}}-\alpha {n}_{{O}_{2}^{+}}$$

3 $$\frac{d{n}_{{O}_{4}^{+}}}{dt}=-\,\gamma {n}_{{O}_{4}^{+}}{n}_{e}+\alpha {n}_{{O}_{2}^{+}}-\eta {n}_{e}$$

4 $$\frac{d{n}_{{O}_{2}^{-}}}{dt}=\eta {n}_{e}$$

These rate equations are simplified by assuming *N*_2_ and *O*_2_ concentrations remain constant throughout the relaxation of the plasma. We substitute the rate coefficients as $\beta =2.1\times {10}^{-8}{T}_{e}^{-0.56}$ for electron-ion recombination, $\eta =3.64\times {10}^{-31}{T}_{e}^{-1}{e}^{-0.052/{T}_{e}}\times {n}_{{O}_{2}}^{2}$ for electron attachment, $\gamma =3.4\times {10}^{-8}{T}_{e}^{-1}$ for recombination with ${O}_{4}^{+}$ and $\alpha =3.4\times {10}^{-8}{T}_{e}^{-1}\times {n}_{{O}_{2}}^{2}+3.3\times {10}^{-35}{T}_{g}^{-3.2}\times {n}_{{O}_{2}}{n}_{{N}_{2}}$ for attachment of ${O}_{4}^{+}$ to neutrals. The electron and the gas temperatures are ${T}_{e}=0.2eV$, ${T}_{g}=0.025eV$, and the Nitrogen and oxygen densities are ${n}_{{N}_{2}}=2.0032\times {10}^{19}\,c{m}^{-3}$, ${n}_{{O}_{2}}=0.5008\times {10}^{19}\,c{m}^{-3}$. Equations 6–8 were solved numerically selecting the initial electro*n* density *n*_*e*_ so that the temporal behaviour of the calculated and the measured density would agree. Equation (9) is not required for the analyses of plasma relaxation and is presented for completeness allowing one to verify that electrical charge is being conserved.

The non-uniform energy distribution over the laser beam cross-section prevented two of the peripheral lens systems from having enough energy to form stable filaments. The *d*_0_ between the remaining eleven convex-concave lens pairs were adjusted to create 11 filaments. The lenses with higher laser beamlet energies were used to create the distant filaments, while the lenses with lower energy input formed the filaments nearer to the lens system (first filament ~150 cm from L_AD_). This energy distribution was selected to preferentiate longer plasma channels. The beams creating the distant filaments are slightly diverging, therefore the more energetic beams could provide a sufficient photon reservoir to sustain the filament for longer distance while in the case of the closer filaments – adding more energy does not increase the length of the channel due to the rapid divergence of the beam after the focus. After establishing 11 stable filaments, the MW diagnostic system was used to measure the plasma density along each filament. Figure 4(c) shows the results of the measurement, where it can be observed that it was possible to generate filaments with densities >10^15^ cm^−3^, over a distance of ~13 m. The filaments closer to the lenses have higher plasma density but are also shorter due to sharper focus^25^. With distance, the filament length increases, while the plasma density is seen to decrease. According to our estimations (see Methods) and simulations of others^30^ the plasma density required to trigger leader propagation is above 10^15^ cm^−3^ therefore this variation in plasma density should not affect discharge initiation though an experimental verification of this is still needed. At distances greater than 1350 cm, it was difficult to establish stable filaments with densities >3 × 10^14^ cm^−3^, the measurement sensitivity threshold of the microwave diagnostic system.

Simultaneous control over a large number of filaments, as in the case described here, requires a good quality of the initial laser beam and in particular high beam uniformity. The energy and intensity contained in each fragment of the beam should be within strict boundaries. On one hand, high energy flux will result in damage to the optical components, on the other hand, higher intensity is required for better control of the remote filaments. Such high power laser beams are typically generated and managed in a vacuum, however, in this case, the beam needs to be extracted into free atmosphere. The extraction of the beam presents an additional limitation on the peak intensity of the profile of the laser beam since damage to the exit window can occur. The optical assembly presented here is constructed of 13 individual telescopic systems. Due to insufficient beam uniformity and the reasons described above, we were unable to achieve sufficient control over 2 of the peripheral lenses and generate controlled plasma filaments. The reason for this is the lack of energy in the peripheral lenses. Increasing the energy of the entire beam was impossible since the most intense areas of the beam damaged the optics. In addition, the use of rectangular beams instead of beams with circular geometry requires additional theoretical and experimental investigation^31^.

### Increasing the lifetime of plasma filaments

We conducted numerical simulations, calibrated in our previous experiments^22–24^, that simulate the conditions required for increasing the lifetime of the plasma filaments with a nanosecond long pulsed laser. The model includes multi-photon and impact ionization, recombination, attachment, detachment and dissociation, electron Joule heating and most importantly gas-dynamic. The electrons are heated by inverse bremsstrahlung and cooled by transferring energy to air molecules and by expansion. The hot electrons excite, ionize and dissociate the molecules. The air is heated by energy transfer from electrons through electronic and vibrational excitation of molecules and cooled due to expansion and heat flow at the boundaries. Cylindrical symmetry is assumed so that the channel is described as a cylinder with radius *R*(*t*) with initial radius of 100 µm. The radial expansion of the hot plasma generates a shock wave in the ambient air.

**Figure 5:**
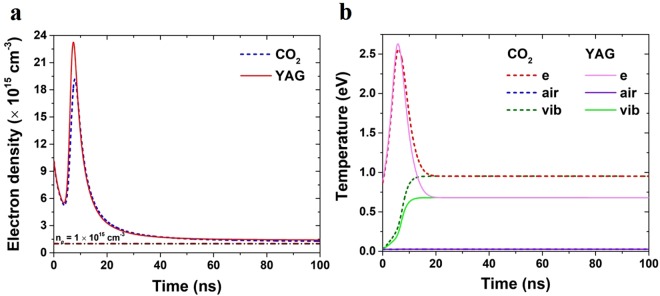
Filament lifetime prolongation simulations. Simulated filament lifetime prolongation by (**a**) CO_2_ (*λ* = 9μm) and Nd:YAG (*λ* = 1.06μm) lasers. (**a**) Free electron density; (**b**) the temperature of the electrons (**e**), the air molecules (air) and the vibrational excitations (vib). The intensity of the Nd:YAG and the CO2 lasers are I_CO2_ = 4.85 GW/cm^2^ and I_Nd_ = 304 GW/cm^2^ with temporal duration of 10 ns. The two pulses have started at the same time.

The long pulse laser-induced electron heating and ionization is accompanied by heating of the neutrals and excitation of molecules and atoms by electron impact, followed by broadband emission from UV to mid-IR. Ionization and excitation rates averaged over a Maxwellian electron energy distribution were used. The code involved 20 species of air plasma and their excited states when important^21^. The code follows separately the evolution of the electron temperature *T*_*e*_ and single air temperature *T*_*air*_ assigned to all molecules, atoms, and ions. This is justified by the fast equilibration time due to their common mass. Within this model, the plasma channel evolution is obtained by solving 24 coupled ordinary differential equations^21^ in time, the electron and air temperatures as shown in Fig. 5, the channel radius and the expansion velocity. These parameters allow for the evaluation of the conductance per unit length of the channel. We calculated the required combination of laser power based on the agreement between experimental data and code predictions as was demonstrated in our previous paper^22–24^. The temporal evolution of the electron density in long broken wire channel obtained by a combination of ultra short and long pulse lasers is presented in Fig. 5(a). The calculations were performed for Nd:YAG as well as for CO_2_ lasers. The intensity required for efficient heating is I_CO2_ = 4.8 GW/cm^2^ and I_Nd_ = 304 GW/cm^2^. The intensity requirement for CO_2_ laser is lower by ~2 orders of magnitude since plasma absorption is more efficient due to the longer wavelength of the laser. The required laser powers are available from a commercial CO_2_ laser using a proper optical delivery system that will provide a capability of illumination of the entire wire. As one can see the plasma channel lifetime at electron densities above 10^15^ cm^−3^ can be extended well above 100 nsec and can lead to the generation of plasma channel conditions for possible propagation of the leader.

The temperature of the electrons, air molecules and the vibration degree of freedom is presented in Fig. 5(b). Normally (i.e. without the second laser) the temperature of the electrons drops on a timescale much shorter than 1 ns, since the energy is being transferred to the vibrational degrees of freedom of the surrounding air^22,23^. In the case of the secondary laser, this energy loss is compensated by additional heating with the secondary laser, sustaining the elevated temperature for much longer times. This elevated electron temperature reduces the relaxation rate of the plasma and prolongs its lifetime.

## Summary and Discussion

We have shown that a 13.5 m long quasi-continuous filament with electron density $> {10}^{15}\,{{cm}}^{-3}$ along the entire distance can be generated using the broken-wire approach. The location of the filaments in space can be controlled individually by manipulating the positions of the diverging lenses in the optical assembly. This opens up the possibility of physically concatenating the filaments limited only by laser pulse energy and profile. The consecutive plasma channels are generated from different fragments of a single high-power femtosecond beam. This implies that all plasma channels are properly timed with respect to each other so that the ionization front propagates with the laser pulse. The natural lifetime of the plasma, (defined as the time for the density to decrease below $5\times {10}^{14}\,{{cm}}^{-3}$) is 3–5 ns. This short recombination time limits the actual length of the high density plasma channel to about a meter since the plasma recombines ~1 m (i.e., 3 ns). A broken wire plasma channel, with a coexisting length of several tens of meters, required for lightning initiation needs to have a substantially longer lifetime.

The lifetime of the plasma filaments can be enhanced above many tens of nanoseconds by heating them using an additional, long pulse beam launched collinearly with the ultrashort pulse^24^. The plasma channel lifetime enhancement measurements were supported by our code that describes the temporal evolution of the plasma channel under the influence of the dual laser pulses irradiation^21^. We used this code to calculate the required intensity for remote heating of the broken wire channel by Nd:YAG and CO_2_ laser. The CO_2_ lasers show greater potential since the required intensity is lower by two orders of magnitude, high atmospheric propagation and lasers with sufficient power are industrially available. Some estimations (see methods) point to a time duration of at least 10 µs^32^. It has been previously shown^21^, in a theoretical work, that the combination of a plasma initiated by a femtosecond filamentation and a long (100 ns) CO_2_ laser pulse can lead to plasma filament lifetime prolongation of much longer than 10 µs. Indeed, additional schemes that rely on much longer mechanisms of discharge initiation, such as, detachment of electrons from O_2_^−^ molecules (generated by plasma filaments) by secondary laser has been shown to reduce the breakdown voltage by ~5%. In that scheme^33^ the resulting electron density is much lower (5×10^11^ cm^−3^) in comparison to our high electron density (>10^15^ cm^−3^) demonstrated by our scheme.

Additional research is required to promote laser assisted lightning control. A possible scheme for the simultaneous broken-wire generation and lifetime prolongation has been proposed in our previous work^24^. Control over laser filamentation at a kilometer range has been experimentally demonstrated^34,35^ though the required accuracy in filament position and shot to shot repeatability of the filament has yet to be verified. Controlling the range and increasing the number of broken-wire fragments will require a different optical setup with larger beam diameter and much greater uniformity of the energy spread along the laser beam.

In conclusion, we have provided a novel scheme of the multi-broken-wire filament and demonstrated the longest air plasma channel, driven by a single femtosecond, terawatt laser. Our ‘multi-broken-wire’ concept produced a 13.5 meter length channel, which is an order of magnitude longer than those achieved in previous studies. The demonstrated concept can be extended to even longer channels using upgraded laser and optical systems. This multi-broken wire concept coupled with filament heating by a second laser pulse is the required missing step that will make remote manipulation of lightning feasible in the near future.

## Methods

### Laser Plasma Triggering Requirements

The plasma channel requirements for discharge triggering have been the subject of many papers^1,7,36–38^. In essence, we require that the laser channel play the role of a long artificial conductor, similar to a long body or wire, which suppresses the applied electric field in the main body and amplifies it at the ends. In the presence of an ambient electric field *E* the linear charge density at a distance *x* from the middle of a channel with length *L* and radius *r* will be given by^7^

5 $${\rho }_{l}=\frac{2\pi {\varepsilon }_{0}Ex}{\mathrm{ln}(L/r)}$$

A key parameter for the feasibility and requirements of laser triggering of lightning is the required channel length *L*. In order to estimate the length *L* we follow Bazelyan and Raizer^7,38^ and rely on the empirical Volt/Ampere relationship characteristic of an air arc at atmospheric pressure for moderate currents observed in a multitude of experiments^39^ given by

6 $${I}_{t}=b/E,b=300\,VA/cm$$

Here *I*_*t*_ represents the threshold value of the current. At this point, we note that the leader velocity *V*_*l*_ can be considered as dependent on the current *I*_*l*_ which flows to the leader tip and feeds it, so that

7 $${I}_{t}={\rho }_{l}{V}_{l}=\frac{2\pi {\varepsilon }_{0}{\rm{\Delta }}U}{\mathrm{ln}(L/r)}{V}_{l}$$

In Eq. (7) we have replaced the channel radius *r* by the effective radius of the leader *R*_*l*_ that encloses the bulk of the charge. For the approximate estimate of the channel requirements that we are addressing here, we take advantage of the empirical relationship that has been established in many laboratory experiments and use the empirical relationship

8 $${V}_{l}\approx a\sqrt{{\rm{\Delta }}U},a=1500\,cm/\text{sec}{V}^{1/2}$$

Equations (5–8) in conjunction with the threshold condition *I*_*l*_ > *I*_*t*_ determine _*t*_hat the threshold length *L*_*t*_ of the plasma channel that ensures the viability of the leader process is given by

9 $${L}_{t}\approx 2{(\frac{b\mathrm{ln}(L/{R}_{l})}{2\pi {\varepsilon }_{0}a})}^{3/2}{(1/E)}^{5/3}$$

Indicates that in the presence of a thundercloud electric field of 1 kV/cm, corresponding to a 10 C cloud charge at 1 km below the centre of a cloud located at a 2 km height, the channel length should exceed *L*_*t*_ = 20 m. The electric field of several kV/cm required for the leader formation^40,41^ is well within the typical fields generated in a thunderstorm^7^. As mentioned in Bazelyan and Raizer^7^, to polarize the plasma channel, a 90 μC charge should flow end-to-end, requiring an average plasma column density of approximately 5 × 10^11^ electrons/cm. For laser produced plasma filaments, this corresponding to plasma density above 10^15^ electrons/cm^3^.

Additional requirement of plasma channel for the efficient guiding of lightning is the lifetime of the plasma. The velocity of the leader propagating along a plasma filament^7^ is estimated to be *Vl* ~ 20 km/s though recent experimental measurements are pointing to a much higher velocity^42^ of *Vl* ~ 2400 km/s. In the last case, the time taken for leader propagation along the distance of *L*_*t*_ is estimated to be *t* ~ *L*_*t*_/*Vl* = 8 µs. Though there is no clarity regarding the exact value of the propagation speed it is clear that a lifetime of several ns (the natural lifetime of plasma filaments) is not sufficient to propagate any significant distance. A substantial lifetime prolongation is required.
